# Inflammation With a Twist: A Rare Urologic Case Report

**DOI:** 10.7759/cureus.109981

**Published:** 2026-05-31

**Authors:** Angela Chen, Jacob Jernigan, Lelia Allamiaghmiouni, Mahmoud Hazim

**Affiliations:** 1 Osteoapthic Medicine, Nova Southeastern University Dr. Kiran C. Patel College Of Osteopathic Medicine, Clearwater, USA; 2 Internal Medicine, Lakeland Regional Health Medical Center, Lakeland, USA; 3 Internal Medicine, UF Health, Jacksonville, USA

**Keywords:** diagnostic methods for testicular torsion, testicular abscess, testicular torsion, testicular torsion management, unilateral orchiectomy

## Abstract

Testicular torsion is a urological emergency that requires prompt recognition and intervention. Although it most commonly affects two age groups, including neonates and adolescents, it can occur at any age. Risk factors to consider include anatomic abnormalities, trauma, testicular tumors, and family history. Typical clinical presentation involves an acute onset of severe scrotal pain, a high-riding testicle, and an absent cremasteric reflex.

We report an unusual case of a 63-year-old man who presented with a two-week history of progressive scrotal pain and swelling and was found to have concurrent testicular torsion and a large scrotal abscess. This atypical and delayed presentation highlights the diagnostic challenges of testicular torsion in older adults.

## Introduction

Testicular torsion results from twisting of the spermatic cord that initially compresses venous outflow through the pampiniform plexus, followed by arterial obstruction and subsequent testicular ischemia. Without timely intervention, prolonged ischemia may result in irreversible testicular damage or loss [1].

Although it most commonly affects two distinct age groups--neonates and adolescents--it can occur at any age [2]. Adult cases are relatively uncommon and account for approximately 14% of all reported cases [3]. Because of this lower prevalence in older adults, torsion is often not immediately suspected, leading to delayed diagnosis.

Torsion may occur spontaneously or in association with precipitating factors such as trauma, strenuous physical activity, or even during sleep [4]. There are some anatomical conditions that increase testicular mobility and susceptibility to twisting along the spermatic cord, most notably the bell clapper deformity. In this condition, inadequate fixation of the posterolateral part of the testis to the tunica vaginalis during embryologic development allows the testis to be placed in a horizontal orientation, predisposing it to intravaginal torsion [5]. Other recognized risk factors include a history of prior torsion, family history, and congenital conditions such as cryptorchidism [4].

The clinical presentation of testicular torsion can be variable. While classic cases involve sudden onset of severe unilateral scrotal pain of less than a 24 hours of duration, often associated with nausea, vomiting, scrotal swelling, and erythema, atypical or subacute presentations can occur [4]. Physical examination findings may include a high-riding testicle (Brenzel sign) and scrotal skin retraction (Ger’s sign) [3]. Because delayed diagnosis is associated with increased rates of complications such as testicular infarction, infertility, and orchiectomy, clinicians must have a high suspicion even in patients with atypical presentations.

## Case presentation

A 63-year-old man with a medical history of hypertension, non-insulin-dependent type 2 diabetes mellitus, hemochromatosis, and prior non-Hodgkin lymphoma status post-partial left lung lobectomy presented with a two-week history of progressive right testicular pain and swelling. He had been hospitalized previously for presumed scrotal infection and treated with intravenous antibiotics, followed by discharge on oral amoxicillin-clavulanate 10 days prior to presentation. Although his pain partially improved, scrotal swelling worsened over the several days preceding admission. He denied fever, chills, night sweats, trauma, hematuria, or urethral discharge but endorsed dysuria. The patient denied hematuria or lower urinary tract symptoms; formal prostate assessment was deferred given the emergent surgical indication.

On presentation, the patient was afebrile, hemodynamically stable, and non-toxic-appearing. Physical examination was notable for an enlarged, tender right hemiscrotum without overlying skin changes or necrosis. Initial laboratory evaluation demonstrated elevated lactic acid, a normal white blood cell count, mildly increased hemoglobin, and decreased platelet levels as summarized in Table 1. No prior platelet count was available for comparison; given the patient's history of prior non-Hodgkin lymphoma and hemochromatosis, chronic thrombocytopenia cannot be excluded, and the etiology remains indeterminate from available data. Renal function and electrolytes were within normal limits.

**Table 1:** Longitudinal trends of key laboratory markers demonstrating postoperative clinical stabilization. Significant findings include the resolution of lactic acidosis, normalization of inflammatory and hematologic indices (white blood cell and platelet counts), and maintenance of hemoglobin stability.

Parameter	On presentation	Post-op day 1	Pre-discharge	Reference range
White blood cell	8.56 x 10^3^/μL	12.48 x 10^3^/μL	5.72 x 10^3^/μL	4.5-11 x 10^3^/μL
Hemoglobin	17 g/dL	14.9 g/dL	13.5 g/dL	13.5-17.5 g/dL (males)
Platelets	72 x 10^3^/μL	70 x 10^3^/μL	77 x 10^3^/μL	150-400 x 10^3^/μL
Lactic acid	4.5 mmol/L	1.3 mmol/L	-	0.5-2.0 mmol/L

**Figure 1:**
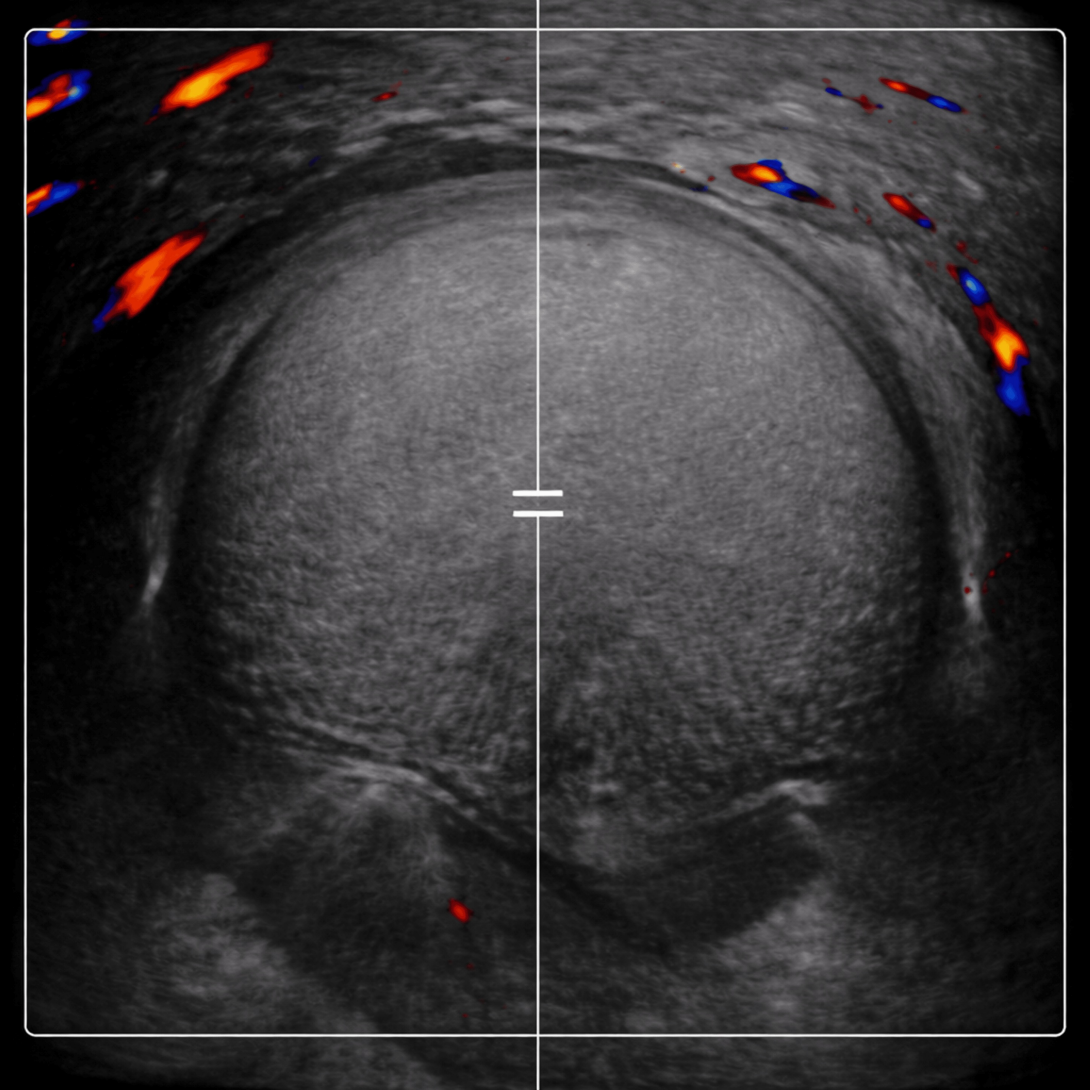
Transverse color Doppler ultrasound of the mid-right testicle demonstrating a complete absence of internal macrovascular flow. While peripheral blood flow is noted in the surrounding scrotal wall, the lack of intratesticular signaling is a hallmark finding of high-grade testicular torsion and global ischemia.

**Figure 2:**
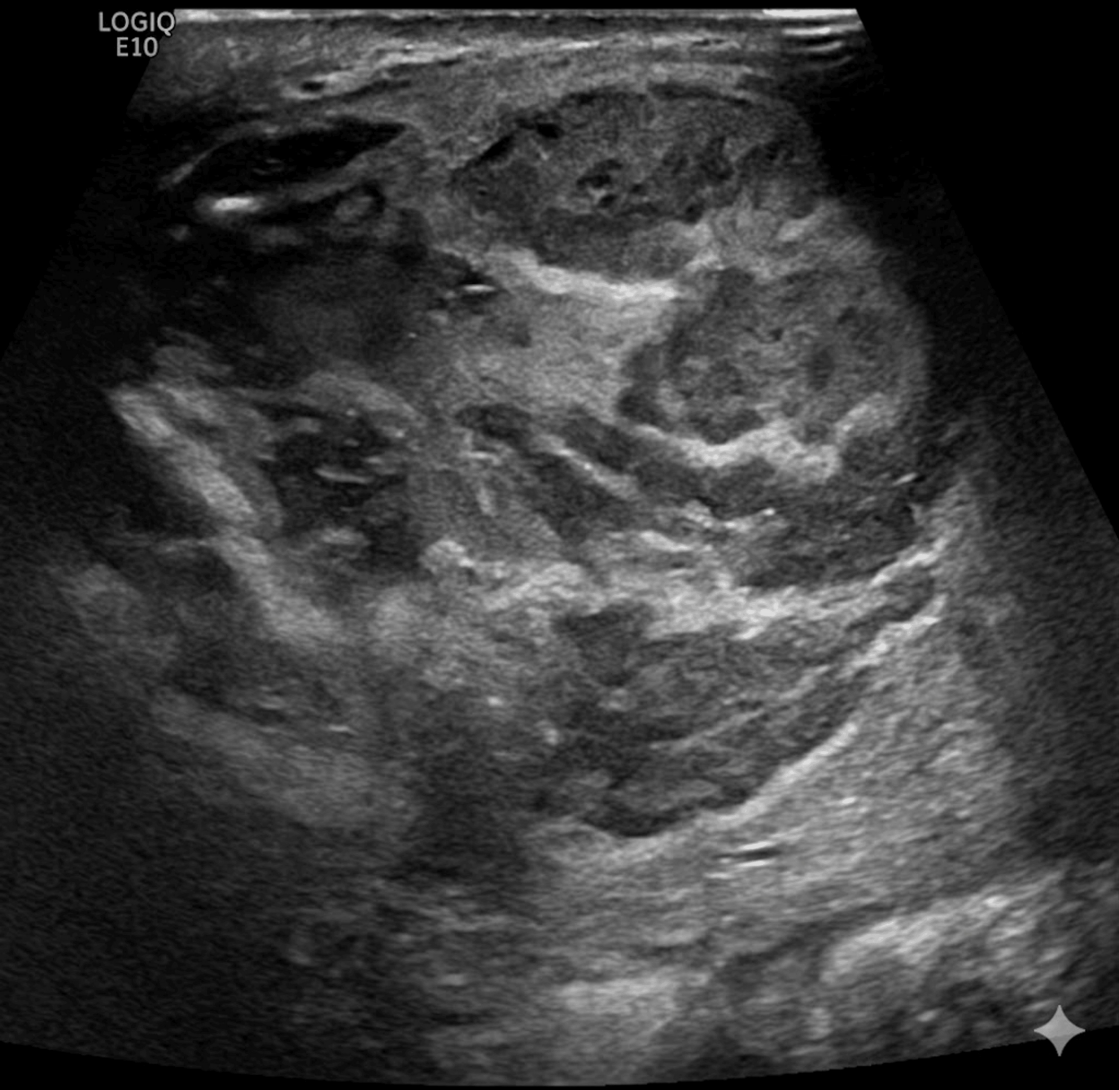
Transverse sonogram of the right scrotum showing a complex, multiloculated fluid collection. The heterogenous appearance and presence of internal echoes are highly suggestive of an abscess. In the context of suspected torsion, these findings likely represent the late-stage inflammatory and necrotic sequelae of prolonged ischemia.

Scrotal ultrasonography revealed a 6.7-cm complex fluid collection in the anterior right hemiscrotum, accompanied by diffuse scrotal wall thickening. Notably, color Doppler imaging demonstrated a complete absence of vascular flow to the right testis (Figures 1 and 2). These findings were highly concerning for testicular torsion complicated by abscess formation. Notably, imaging was obtained concurrently with surgical consultation and did not delay operative intervention. Given the surgical urgency, urology was consulted, and the patient was taken emergently to the operating room for exploration.

Upon incision and drainage via a transscrotal approach, a large volume of foul-smelling purulent material was encountered, and specimens were obtained for microbiologic culture. Intraoperatively, the right testicle was found to be densely adherent to the surrounding scrotal wall and adjacent soft tissues. The testis appeared markedly discolored with a green hue, consistent with prolonged ischemia and non-viability. A right orchiectomy was performed, and a Penrose drain was placed. The patient tolerated the procedure well with multimodal pain management.

Postoperatively, the white blood cell count increased on day 1 before decreasing by the day prior to discharge (Table 1). Empiric broad-spectrum antibiotics were initiated and later narrowed to ertapenem after cultures grew extended-spectrum beta-lactamase-producing *Escherichia coli*. A peripherally inserted central catheter was placed for outpatient antimicrobial therapy. The patient was discharged in stable condition six days after the procedure with well-controlled pain and scheduled urology follow-up within two weeks.

## Discussion

Testicular torsion remains one of the most time-critical diagnoses in clinical medicine [1]. While its classic bimodal distribution highlights neonates and adolescents, adult cases comprise approximately 14% of the total incidence [3, 5]. In the geriatric population, diagnostic delay is the primary driver of high orchiectomy rates, often rooted in diagnostic anchoring where clinicians favor more prevalent conditions such as epididymo-orchitis, incarcerated hernias, or malignancy over primary ischemia [2, 6]. Recent reports of torsion in patients aged 57 and 79 underscore that age provides no immunity and that clinicians must maintain a high index of suspicion even when the clinical picture is atypical [7, 8].

The patient's two-week symptomatic progression is a striking deviation from the traditional 6-12-hour golden window for salvage [1]. This case highlights a dangerous diagnostic trap involving the initial use of amoxicillin-clavulanate. In subacute presentations, antibiotics may provide a transient, partial dampening of the secondary inflammatory response, creating a false sense of clinical improvement [6]. Critically, this is evidenced by the patient's own clinical trajectory--partial pain improvement alongside concurrent worsening of scrotal swelling--a discordant response reflecting inflammatory suppression without treatment of the underlying ischemic process. This pharmacological masking further delays surgical intervention until irreversible necrosis occurs [6]. Two potential pathophysiologic mechanisms may explain this presentation. One possibility is that primary testicular torsion led to ischemia and necrosis, followed by secondary infection and abscess formation. Alternatively, severe epididymo-orchitis may have caused intense inflammation within the inelastic tunica albuginea, creating a compartment syndrome effect where increased intratesticular pressure obstructs venous outflow and leads to secondary infarction [6]. Clinicians should be aware that overlapping infectious features such as scrotal erythema, swelling, and purulent discharge can lower suspicion for torsion; however, an absent cremasteric reflex and complete absence of intratesticular flow on color Doppler--in contrast to the preserved vascular signal typical of inflammatory conditions--remain the most reliable differentiating features in this clinical scenario. Given the intraoperative findings of a green hue and dense adhesions, a primary ischemic event leading to late-stage necrotic transformation is the more likely etiology [4, 6, 7]. It should be emphasized that once torsion was suspected on this admission, there was no in-hospital delay; prompt ED evaluation with scrotal ultrasonography demonstrating absent intratesticular flow led to immediate urologic consultation and emergent operative intervention.

The clinical imperative for salvage in geriatric patients extends beyond reproductive potential to the preservation of endocrine function. Research indicates that unilateral orchiectomy can significantly reduce serum testosterone levels even when the contralateral testis is healthy [9]. Post-orchiectomy, the endocrine system may enter a state of dynamic disorder characterized by increased follicle-stimulating hormone and luteinizing hormone levels alongside a decrease in testosterone that often fails to recover to baseline [9]. In older males, this surgical reduction in androgen production can exacerbate age-related hypogonadism, contributing to accelerated bone density loss, metabolic syndrome, and cognitive decline [9].

Despite the complexities of a geriatric presentation and the presence of a 6.7-cm multiloculated abscess, color Doppler ultrasound remains the gold standard for identifying the absence of vascular flow [10, 11]. Beyond imaging, a meta-analysis by Yu suggests that familial patterns of inheritance can contribute to torsion risk, indicating that a family history of the condition should further heighten clinical suspicion regardless of age [12]. Ultimately, surgical exploration should not be delayed for imaging if clinical suspicion is high, as the degree of torsion and the duration of ischemia are the final determinants of viability [13]. This case reinforces that any presentation of acute or subacute scrotal swelling in an older adult must be treated as torsion until proven otherwise, as early surgical exploration remains the only intervention capable of improving outcomes [7, 8].

It should be noted that contralateral orchiopexy, which is standard of care following unilateral testicular torsion to prevent future torsion of the remaining testis, was not performed at the index procedure; the reasoning for this omission was not documented and represents a limitation of the reported management.

## Conclusions

This case describes a rare but clinically significant presentation of concurrent testicular torsion coexisting with a large scrotal abscess in an older adult male. It underscores the need for clinicians to maintain a high suspicion for testicular torsion even in patients outside the typical adolescent age group and in non-classic presentations that overlap with infectious processes. Prompt imaging and emergent urologic consultation remain essential to ensuring timely diagnosis and management.
